# Spatial Overlap and Behavioral Interactions Among Four *Habronattus* Jumping Spider Species in a Mixed‐Species Assemblage

**DOI:** 10.1002/ece3.70871

**Published:** 2025-03-30

**Authors:** Jenny Y. Sung, Jack A. Fogle, Nathan I. Morehouse

**Affiliations:** ^1^ Department of Biological Sciences University of Cincinnati Cincinnati Ohio USA; ^2^ Laboratory of Neuroethology Sokendai‐Hayama (The Graduate University for Advanced Studies), Shonan Village Hayama Japan

**Keywords:** behavioral ecology, courtship, habitat use, interspecific interactions, reproductive interference, space use

## Abstract

Within mixed‐species assemblages, closely related species may face interspecific competition and reproductive interference. Species may evolve adaptations to outcompete or coexist, though most assemblages likely exist in a nonequilibrium state between the two extremes. Understanding the dynamics between potentially syntopic species requires careful studies conducted at a fine spatial resolution, an especially important consideration for small invertebrate animals. We characterized interspecific interactions within a mixed‐species assemblage consisting of four species of *Habronattus* jumping spiders, a genus that can be found in syntopic assemblages where males are known to indiscriminately court conspecific and heterospecific females alike. Through detailed focal observations, we evaluated how species overlap in their spatial occurrence, temporal occurrence, light environment, movement level, and behavioral interactions, including courtship. We assessed whether the observed heterospecific interactions occurred more frequently than random chance by comparing the observed data to network reference models. Our findings revealed interspecific overlaps across all evaluated variables, which may indicate shared environmental requirements amongst the species and potential interspecific competition and interference. However, there was a notable temporal shift between *H. calcaratus* and *H. coecatus* . *H. decorus* showed some divergence in spatial occurrence and light environment that may be a phylogenetic signal or potentially associated with its distinct courtship display. Additionally, we observed sex differences in movement levels across all species that could represent a selection for male “scramble competition” strategy. Lastly, heterospecific behavioral interactions, including courtship, were frequently observed but occurred less often than random chance, as represented by network reference models. Altogether, our findings suggest that individuals in this assemblage are engaged in some level of interspecific competition and reproductive interference, but there may be mechanisms to mitigate these pressures. This study establishes an ecological foundation to investigate possible adaptive responses in this assemblage, such as reproductive character displacement and sensory drive.

## Graphical abstract

Closely related, syntopic species may face interspecific competition and reproductive interference, but current tracking technology do not provide enough fine‐scale spatial resolution to adequately evaluate the interspecific dynamics of small invertebrate animals. Through focal observations, we found four species of *Habronattus* jumping spiders to exist in syntopy, overlapping in their space use, temporal occurrence, light habitat preference, and behavioral interaction (including frequent heterospecific courtship), though some species showed habitat and behavioral divergence, and network modeling comparisons showed that heterospecific interactions in this assemblage occurred less often than chance. Altogether, our findings suggest that species in this mixed‐species assemblage are engaged in some level of interspecific competition and reproductive interference but may be evolving mechanisms to mitigate these pressures.

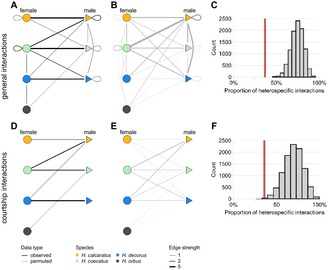

## 1 Introduction

Closely related species that co‐occur within the same environment may also compete in the use of limited resources in the environment (e.g., habitat, food). Direct resource competition between species can lead to local extinctions of the less competitive species or, alternatively, cause species to diverge in their niche or other traits to reduce competition (de Bello et al. 2021; Hardin 1960; MacArthur and Levins 1967). Another possible effect of direct species interactions is reproductive interference, wherein interspecific interactions result in reproductive fitness costs to at least one of the species involved (Burdfield‐Steel and Shuker 2011; Gröning and Hochkirch 2008). These costs can be direct (e.g., gametic investment during maladaptive hybridization events) or indirect (e.g., energetic expenditures during misdirected courtship and mating attempts). The latter can be especially costly for animals with energetically expensive courtship displays (Cady, Delaney, and Uetz 2011; Gröning and Hochkirch 2008; Myers et al. 2013; Wedell 2010). Similar to resource competition, there may be selection for differences in niche, traits, as well as behaviors that reduce reproductive interference and/or facilitate conspecific mating success (e.g., reinforcement and reproductive character displacement; Gröning and Hochkirch 2008; Höbel and Gerhardt 2003).

In reality, most cases of syntopic species assemblages likely fall somewhere in the middle of a spectrum of nonequilibrium states between the extremes of coexistence and direct competition/interference, in which case species would exhibit some levels of competition or interference as well as varying levels of habitat or behavioral segregation. Such nonequilibrium states can be observed across diverse taxa such as birds, snakes, mammals, insects, and spiders (Davies et al. 2007; Hochkirch, Bücker, and Gröning 2008; Singer 1990; Steen et al. 2014; Taylor, Powell, and McGraw 2017; Yackulic et al. 2014). Alternatively, related species within the same habitat may exhibit no interspecific competition, interference, nor adaptations to reduce these potential conflicts, and any observed diversity between species habitat use and adaptive traits may be attributed to more stochastic processes such as dispersal and evolutionary events (e.g., speciation and extinction), as described by neutral theory (de Bello et al. 2021; Hubbell 2001).

Unveiling the dynamics of interspecific interactions within mixed‐species assemblages requires careful observations of how species engage with the habitat and each other at a “fine” spatial scale (Wiens 1989). For example, in a classic niche partitioning study, five *Dendroica* warbler species were found to occupy the same tree species within their shared habitat. However, closer inspection revealed that each species spatially segregated to distinct regions of the trees and specialized in different food sources (MacArthur 1958). Species may also diverge in their temporal occurrence within a shared habitat, such as by seasonal or diel patterns of activity (e.g., Lear et al. 2021; Tauber and Tauber 1976), or by even more subtle temporal partitioning during behavioral interactions (e.g., species can differentiate in the timing of their calls on a fine timescale to avoid acoustic overlap; see Allen‐Ankins and Schwarzkopf 2021 and Hart et al. 2021). The habitat itself may also be heterogeneous (e.g., light levels and substrate types) and affect signal propagation and signal perception, and species may differentially occupy these microhabitats in ways that increase intraspecific communication and/or reduce interspecific competition and interference (e.g., Elias, Mason, and Hoy 2004; Leal and Fleishman 2002; Rosenthal et al. 2019). Collectively, these studies underscore the importance of conducting observations at behaviorally relevant scales when evaluating interspecific interactions, competition, and coexistence. This is a particularly key issue for small but active invertebrate animals, such as jumping spiders, whose movements through the environment and behavioral interactions with conspecifics and heterospecifics occur at much finer spatial resolutions than can be captured using common telemetry and tracking technologies.

*Habronattus* jumping spiders (Araneae: Salticidae) offer an interesting opportunity to investigate interspecific dynamics within congeneric species assemblages. This genus consists of more than 100 species distributed primarily throughout North America, where species can be found in single species populations as well as mixed‐species assemblages (Brandt et al. 2020; Griswold 1987; Hill 2012; Maddison and Hedin 2003; Taylor, Powell, and McGraw 2017; Taylor, Cook, and McGraw 2019; Wayne P. Maddison, personal communications; personal observations, JYS). Focal follow studies have provided key understanding of how these animals interact with the environment and with each other (e.g., Blackburn and Maddison 2015; Brandt, Kelley, and Elias 2018; Miller 1971; Scheidemantel 1996). Recent work by Taylor and colleagues indicated that syntopy in one mixed‐species assemblage involves some partitioning of microhabitat usage as well as interspecific behavioral interactions, including misdirected courtship (Taylor, Powell, and McGraw 2017). Their findings suggest that this assemblage may face some levels of reproductive interference due to misdirected courtship attempts by males toward heterospecifics. Hybrid mating was not directly observed in the study by Taylor et al., but hybridization events are known to occur with some frequency in this genus based on morphological, ecological, and phylogenetic evidence of introgression events amongst several lineages within this genus (Bougie, Brelsford, and Hedin 2024; Hedin and Lowder 2009; Leduc‐Robert and Maddison 2018; Maddison and McMahon 2000). Additionally, hybridization in this group may be partially driven by sexual selection, as seen in the preference of female *H. pugillis* for novel and unfamiliar traits in males (Elias, Hebets, and Hoy 2006; Hebets and Maddison 2005; Masta and Maddison 2002).

**FIGURE 1:**
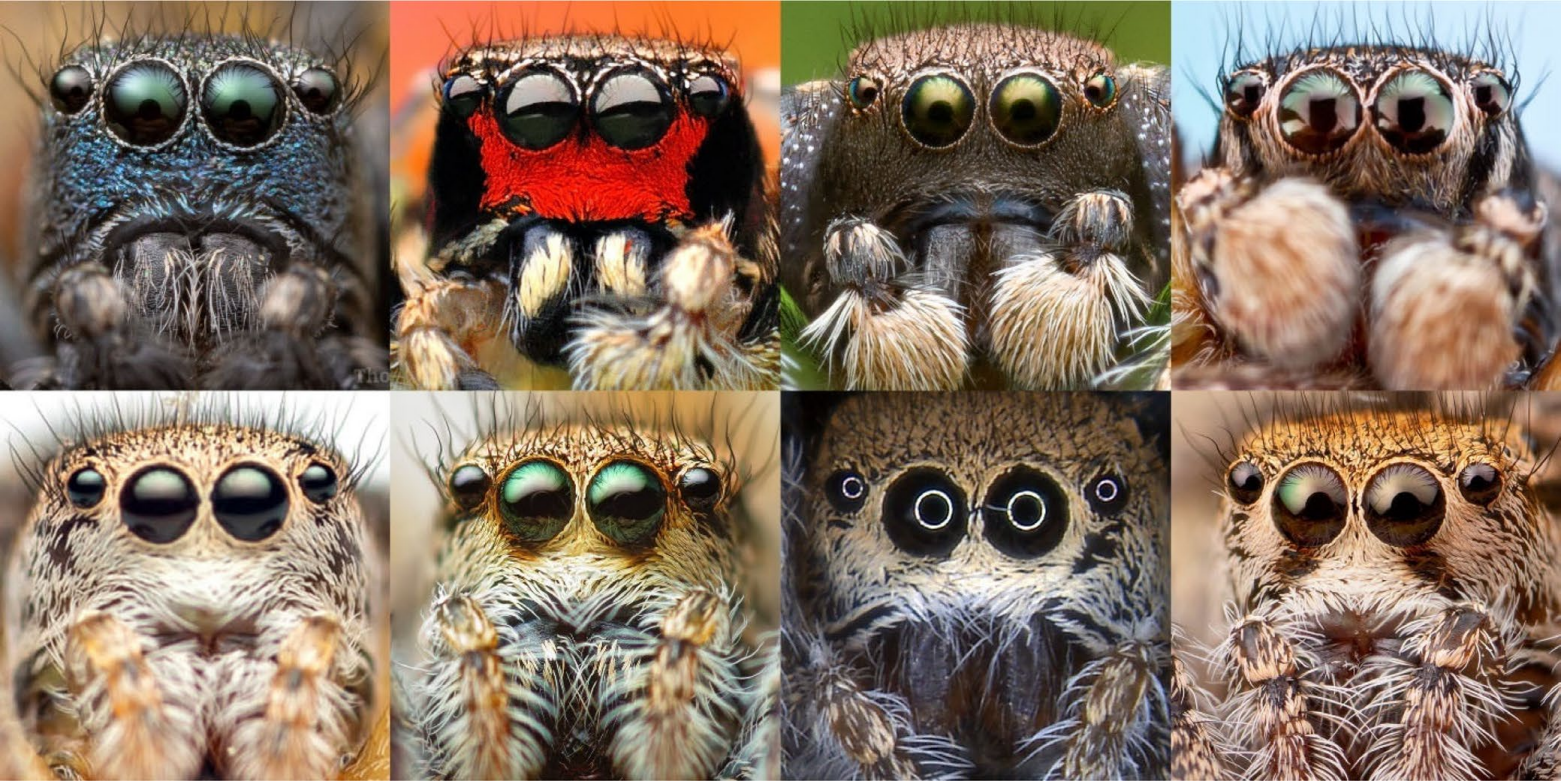
Faces of male (top) and female (bottom) *Habronattus* species found within the focal mixed‐species assemblage at the Edge of Appalachia Preserve. Across species, male faces show diverse colors and patterns while female faces are tan with less interspecific distinctions. Species are arranged in order of phylogenetic relatedness (from left to right: *H. decorus, H. coecatus, H. calcaratus*, and *H. orbus* ), with *H. decorus* being the least phylogenetically related to other species. Photo credits (left to right, top to bottom): Thomas Shahan, Bao Xuan Ngo, Patrick Zephyr, Thomas Shahan; Tiziano H.‐C., Thomas Shahan, Jenny Sung, Thomas Shahan.

In this present study, we characterized the environmental overlaps and behavioral interactions of four species of widely distributed *Habronattus* spiders found in a mixed‐species assemblage at the Edge of Appalachia Nature Preserve, Ohio (Figure 1). This area offers a variety of diverse habitats, endemic species, and rare communities juxtaposed between distinct ecosystems of modern Appalachian forests and midwestern prairies (Bedel 2012; Braun 1928; Strittholt and Boerner 1995). *Habronattus* species at this site have previously been sighted to be in close proximity to each other, but interspecific interactions have not been formally evaluated. Using a focal observation approach, we collected indirect observations of species interactions (i.e., overlap and similarities in spatial occurrence, temporal occurrence, light environment, and movement activities across the habitat) and direct observations of intra‐ and interspecific behavioral interactions. We discussed our findings in the contexts of interspecific competition, habitat segregation, and reproductive interference in this species assemblage, specifically highlighting misdirected heterospecific courtships.

## 2 Materials and Methods

### 2.1 Field Site and Study Species

**FIGURE 2:**
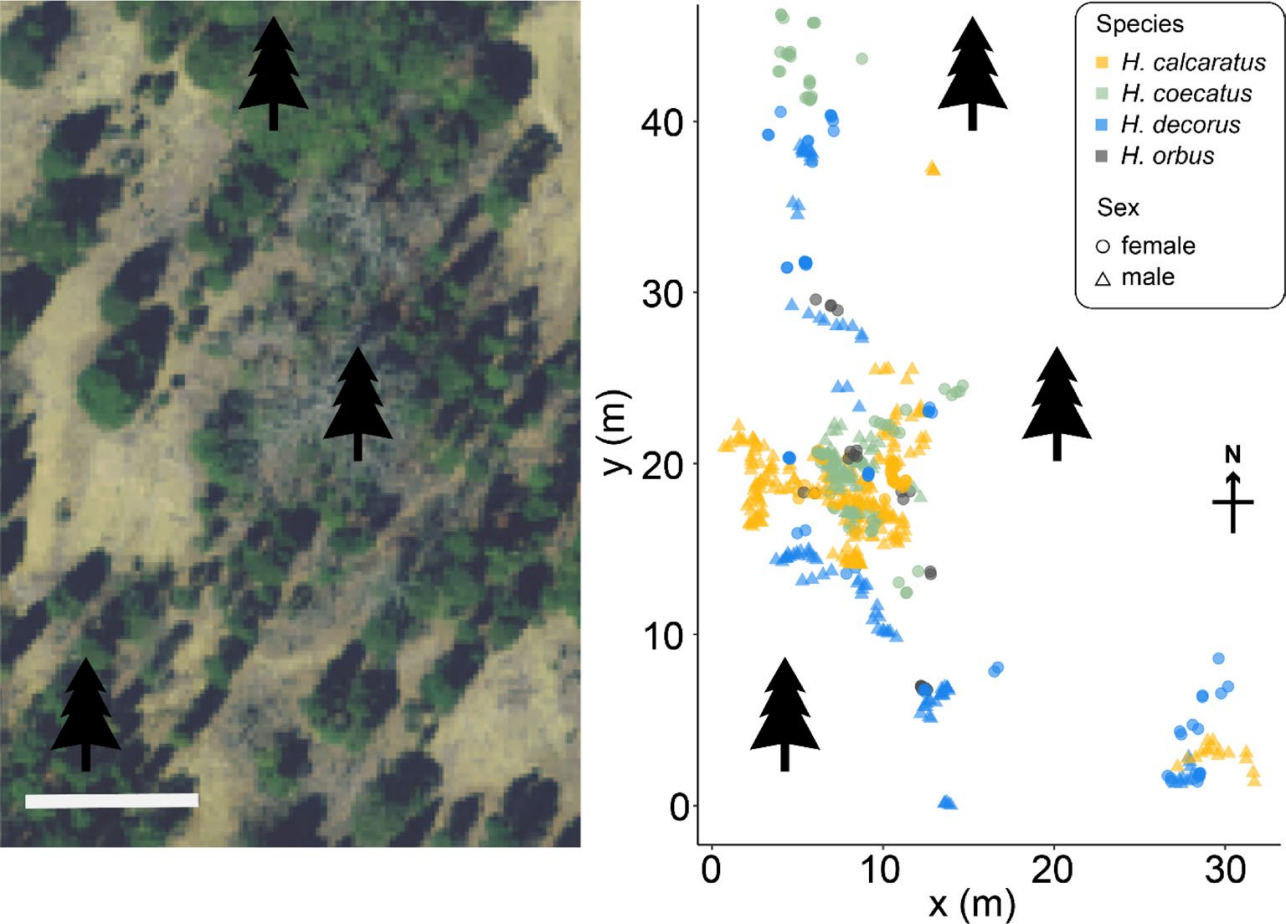
Aerial view of study site (left; Map Data 2022 Google) and spatial occurrences of focal *Habronattus* spider species plotted onto cartesian coordinates (right). For spatial data, color represents species, and shape represents sex (see inset legend on right). *Habronattus* spiders in their natural habitat at the Edge of Appalachia Nature Preserve were observed in open areas and adjacent grassy areas, excluding heavily forested areas. White scale bar indicates 10 m (left). Black tree symbols provide comparative reference points between the two maps.

We evaluated four *Habronattus* species ( *H. decorus* , *H. coecatus* , *H. calcaratus* , and *H. orbus* ; Figure 1) within a shared species assemblage at the Edge of Appalachia Nature Preserve in West Union, Ohio, USA (Adams County, 38°45′48.472″ N, 83°27′37.341″ W). The study site is classified as humid continental climate in the Köppen‐Geiger classification system, and its general habitat consists of cedar barrens surrounded by pine forests. The area of the preserve used for observations was in a restricted area that received minimal human disturbances. Permission to conduct this study was granted by the Cincinnati Museum Center and The Nature Conservancy. The general region for this study was selected based on previous observations of species occurrences at the site (David Outomuro and David J. Morris, personal communications) and habitat descriptions of where each species commonly occurs at sites across the US (Wayne P. Maddison, personal communications). Since the methods used to collect spatial data required physical measurements against set landmarks in the environment (described below), spider observations needed to occur within a defined space of the habitat that could represent where all four species naturally occurred. To define the specific region used for this study, we first surveyed two broad areas of approximately 1995 m^2^ between March and May of 2021, including all substrate types (e.g., open, barren area, pine forests, and sparsely grassy areas). Based on the locations where spider were observed to occur during this time period, we focused our search efforts for spider observations within an area of approximately 1050 m^2^ (Figure 2, left). This focal area served as the area in which observers began searching for spiders, but it did not define any spatial limit on how far a spider could travel during an observation (i.e., no observation ended prematurely due to a spider passing a predefined spatial boundary).

The *Habronattus* phylogeny is relatively well‐resolved, and of these species, *H. calcaratus* and *H. orbus* are the most closely related species (“*viridipes*” species group), whereas *H. coecatus* and *H. decorus* are placed in distinct subgroups within the genus (“*coecatus*” and “DTB” (*decorus*/*texanus*/*banksi*) species groups, respectively; Leduc‐Robert and Maddison 2018; Maddison and Hedin 2003). The three former species, excluding *H. decorus*, also belong in the larger “VCCR” (*viridipes*/*coecatus*/*clypeatus*/*roberti*) clade (Leduc‐Robert and Maddison 2018).

### 2.2 Spider Observations

Spiders were observed in their natural habitat between March and August of 2021 and 2022 during daylight hours (0900–1830 h). A total of approximately 302 person‐hours were spent searching for and observing spiders across 19 days. During the period of peak spider abundance, observations were conducted at least once per week, excluding periods with conditions in which spider sighting and activities were unlikely (e.g., precipitation, extreme temperatures). Each search was initiated from a random spot within the 1050 m^2^ focal area, including open and grassy substrate types, by slowly walking the entirety of this region and scanning the substrate until a spider is found. Once a spider was found, we conducted a 15‐min focal follow observation.

During each observation, we collected data on the spatial occurrence, temporal occurrence, light environment, movement level, and behavioral interactions of the focal spider. This study focused on interactions between reproductively mature individuals, but penultimate subadult spiders (henceforth referred to as “subadults”) were included because some mature spiders actively interacted with them during our observations. Verbal notes were taken using an audio recorder, and observations were conducted in pairs to ensure that spider behaviors were not missed. Observers minimized potential disturbances by limiting movements and sounds, maintaining a distance of about 1 meter. In this manner, spiders either showed no reaction or briefly oriented toward the researcher for a few seconds before returning to their original position (personal observations, JYS; see also Blackburn and Maddison 2015; Taylor, Powell, and McGraw 2017). During an observation, observers would follow the spider if the spider moved. After the observational period, spiders were caught to confirm their species, sex, and maturity. Then, each spider was marked on the ventral side of the abdomen with liquid eyeliner to allow for the identification of unique individuals. There were at least five observations of repeat spiders, which were discarded and excluded from analyses. Additionally, neighboring spiders that were found in the focal spider's vicinity during observations (“attendees”) were collected once the behavioral interaction(s) concluded or if the attendee spider had moved away from the focal spider. In total, spanning all datasets, 108 individuals were observed, composed primarily of mature spiders (*n* = 94) and a small subset of penultimate subadult spiders (*n* = 14). With the exception of the temporal data, the sample sizes for each following subsections were a subset of this total sample size due to methodological constraints (i.e., the spatial and movement datasets excluded trials that were incomplete and less than 15 min, and light environment dataset excluded some trials conducted during cloudy conditions; details are explained in each respective section below).

### 2.3 Site Mapping and Spatial Measurements

To investigate how this assemblage of potentially syntopic *Habronattus* species interact with each other spatially, we first created a local map. Since jumping spider movements are too fine‐scaled to utilize passive tracking or GPS, we created a local map by triangulating distances and angles between multiple local landmarks across the observation site. Then, we tracked spider movements with direct, manual measurements: during observations, a flag was planted for each spot where spiders lingered or changed direction while traveling. After each observation, we measured the distance and cardinal direction of each flag against our local landmarks. Final spatial data were assembled using Adobe Illustrator, and *x*–*y* coordinates were exported to calculate the amount of spatial overlap between species. Spatial data collection was not replicated in other mixed‐species assemblages.

### 2.4 Spatial and Temporal Occurrence

We evaluated overall space usage, spatial overlap, and phenology of each species and sex. Spatial data from 84 unique individual spiders were evaluate: *H. calcaratus* female (*n* = 7), male (*n* = 16); *H. coecatus* female (*n* = 18), male (*n* = 8); *H. decorus* female (*n* = 18), male (*n* = 11); *H. orbus* female (*n* = 6). We estimated the kernel utility distribution (KUD) based on 50% and 95% space usage to represent each species' “core home range” and “extended home range,” respectively. We used this to compare spatial overlap by calculating the proportion of home range overlap (HR_i,j_) between every species pair with the following function,

$${\mathrm{HR}}_{i,j}=A_{i,j}/A_{i}$$

wherein *A*_*i,j*_ is the area of overlap shared between species *i* and species *j*, and *A*_*i*_ is the area of home range for species *i* (Calenge 2006). We also looked at KUD estimations and space overlap at the species*sex level using the same approach, but there were no obvious patterns (Figure S2). All data analyses and visualizations in this section and beyond were created using R (R Core Team 2024 [versions 4.1.0 and 4.4.0]; RStudio Team 2020 [version 2023.12.1.402]). Spatial data were analyzed using the package “adehabitatHR” (Calenge 2006).

For phenology, we noted the date and time of day of each spider's observation. Over the 2021 and 2022 field seasons, phenology data were collected for all 108 spiders: *H. calcaratus* female (*n* = 9), male (*n* = 21); *H. coecatus* female (*n* = 21), male (*n* = 14); *H. decorus* female (*n* = 21), male (*n* = 13); *H. orbus* female (*n* = 9). To account for unevenness and clustering in sampling days, we applied a “geom” smoothing function with the locally weighted scatterplot smoothing method in the package “ggplot2” (Wickham 2016).

### 2.5 Light Environment and Movement Level

To understand how *Habronattus* spiders might differentially utilize the environment, we observed the (1) light environment and (2) movement level of each spider. Light environment was defined as the amount of time each spider spent in a sunny vs. shaded environment during the observation. Light environment was quantified as a relevant microhabitat because prior research indicated that *Habronattus* color vision can become compromised in low light or shaded conditions (see Taylor and McGraw 2013). Thus, the level and quality of light may be an important environmental factor that influences color‐based foraging and other behavioral interactions. Light environment was quantified bimodally, in which spiders were considered to be in the sun or under the shade of a local environmental feature (e.g., grass blade, stick). In moving spiders, the observer noted the time point at which spiders transitioned between the two environments. These quantifications were made to the nearest second. Light environment data was not quantified during periods of overcast conditions due to the lack of clear, bimodal distinction between what may be considered sun vs. shade conditions as clouds passed overhead. We collected light environment data of 88 individuals (78 adults, 10 subadults): *H. calcaratus* female (*n* = 8), male (*n* = 17); *H. coecatus* female (*n* = 16), male (*n* = 10); *H. decorus* female (*n* = 20), male (*n* = 11); *H. orbus* female (*n* = 6).

Movement levels were measured as the linear distance traveled by an individual spider during observations (i.e., distances between the flags used to gather spatial data). Movement data were collected for 69 individuals (57 adults, 12 subadults): *H. calcaratus* female (*n* = 7), male (*n* = 14); *H. coecatus* female (*n* = 15), male (*n* = 8); *H. decorus* female (*n* = 12), male (*n* = 8); *H. orbus* female (*n* = 5). Differences in both light habitat and movement levels were calculated using the Kruskal–Wallis test, followed by multiple comparisons using Dunn's test with Bonferroni correction (using the package "FSA"; Ogle et al. 2023). We conducted the Scheirer‐Ray Hare test to evaluate the effects of species and sex using the package “rcompanion” (Mangiafico 2024).

### 2.6 Behavioral Interactions

For behavioral interactions, we noted the species, sex, and maturity of interacting spiders, and we determined the type of behavior as (a) looking (i.e., spider reoriented to look), (b) courtship (i.e., male courtship display, mating), or (c) aggression (i.e., antagonistic display, attack). Of the 105 focal spiders we observed, 61 were found in close vicinity (i.e., 0.5 m or less) to a mature or subadult congener (of the 61 spiders, 15 were subadults). A total of 33 pairs of behavioral interactions were observed between 63 focal and attendee spiders, including three male spiders that interacted with more than one spider ( *H. calcaratus* female *n* = 8, male *n* = 14; *H. coecatus* female *n* = 15, male *n* = 9; *H. decorus* female *n* = 7, male *n* = 8; *H. orbus* female *n* = 2; of these, eight were subadults). Behavioral interactions were evaluated in two ways. First, we considered the full dataset with all behaviors included. Second, we separately analyzed the subset of interactions that involved courtship (14 courtship interactions between 25 spiders, with three male spiders courting multiple females: *H. calcaratus* female *n* = 2, male *n* = 5; *H. coecatus* female *n* = 6, male *n* = 3; *H. decorus* female *n* = 5, male = 3; *H. orbus* female *n* = 1). These two sets of interaction data were visualized as networks using the “igraph” R package (Gábor and Nepusz 2006). Next, for each network, we calculated the percentage of heterospecific interactions by dividing the number of heterospecific interactions over the total number of interactions, such that 100% signifies a network showing only heterospecific interactions, and 0% signifies a network showing only conspecific interactions.

Finally, to ask whether the observed percentage of heterospecific interactions were more or less likely than expected based on random chance, we used a permutation‐based method to create reference models based on the interacting individuals found within our *Habronattus* assemblage. We retained the vertices of our observed dataset (i.e., the identities of each spider involved in a behavioral interaction) and randomized the edges (i.e., connections between vertices) 10,000 times in R. Seeds were set as “1” through “10,000.” Permutations were conducted for both the “all interactions” and “courtship only” networks. For each reference model, we summarized the result as a distribution of each permutation's percentage of heterospecific interactions. This permutation‐based distribution was then used to calculate the probability P of observing our actual results in an assemblage defined by random interactions with regard to species identity. Thus, we were able to compare our observed results to three interaction models: a completely random‐interaction model (which represents the absence of bias toward interacting with conspecifics over heterospecifics), a heterospecific‐only model (100% heterospecific interactions), and a conspecific‐only model (0% heterospecific interactions).

## 3 Results

### 3.1 Spatial Distribution and Overlap

The local habitat where our focal *Habronattus* species were found was composed mostly of open ground surfaces with pebbles and sparse grass, including some transitional areas connecting to pine forests (Figure 2). Spiders were readily observed in the grassy and open areas, but no spiders were observed within the pine forest undergrowth. Curiously, while female *H. orbus* were observe at this site, males were not found during the study, although they were seen at other locations at the preserve. This may be due to slight differences in microhabitat availability at different sites (i.e., male *H. orbus* may prefer leaf litters not present at our observation site; Wayne P. Maddison, personal communications).

**FIGURE 3:**
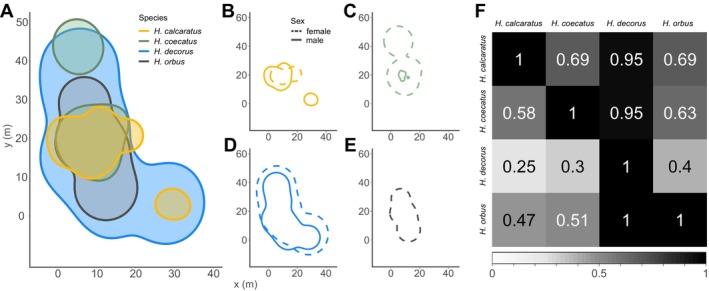
Extended home range (estimated 95% space usage) and spatial overlap of all four species (A). Color represents species. The extended home range is shown for each species and sex: *H. calcaratus* (B), *H. coecatus* (C), *H. decorus* (D), and *H. orbus* (E). In the subplots (B–E), line style represents sex. Overlap values (F) between each species pair (e.g., sp_*i*_ and sp_*j*_) were calculated by the amount overlap between the species pair divided by the total home range of the species represented in the row (overlap_*i*,*j*_/HR_*i*_). A value of 1 denotes complete overlap, and 0 denotes no overlap.

The extended home range (estimated 95% space use) of *H. calcaratus* covered 363.6 m^2^, *H. coecatus* covered 431.3 m^2^, *H. decorus* covered 1357.4 m^2^, and *H. orbus* covered 536.4 m^2^. All four species' extended home ranges spatially overlapped with each other (Figure 3A). Females and males also overlapped within each species (Figure 3B–E). *H. calcaratus* , *H. coecatus* , and *H. orbus* all showed similar overlap values with each other (Figure 3F). However, *H. decorus* showed very different patterns from the other species (high overlap when compared to the total space use of the opposite species, but low overlap with all other species when dividing by their own space use; Figure 3F). This is likely due to the larger size of their extended home range. The core home range (estimated 50% space usage) data showed a similar pattern as seen in the extended home range of each species (Figure S1). When examining the spatial overlap between species and sex, there were no clear pattern (Figure S2).

### 3.2 Temporal Distribution and Overlap

**FIGURE 4:**
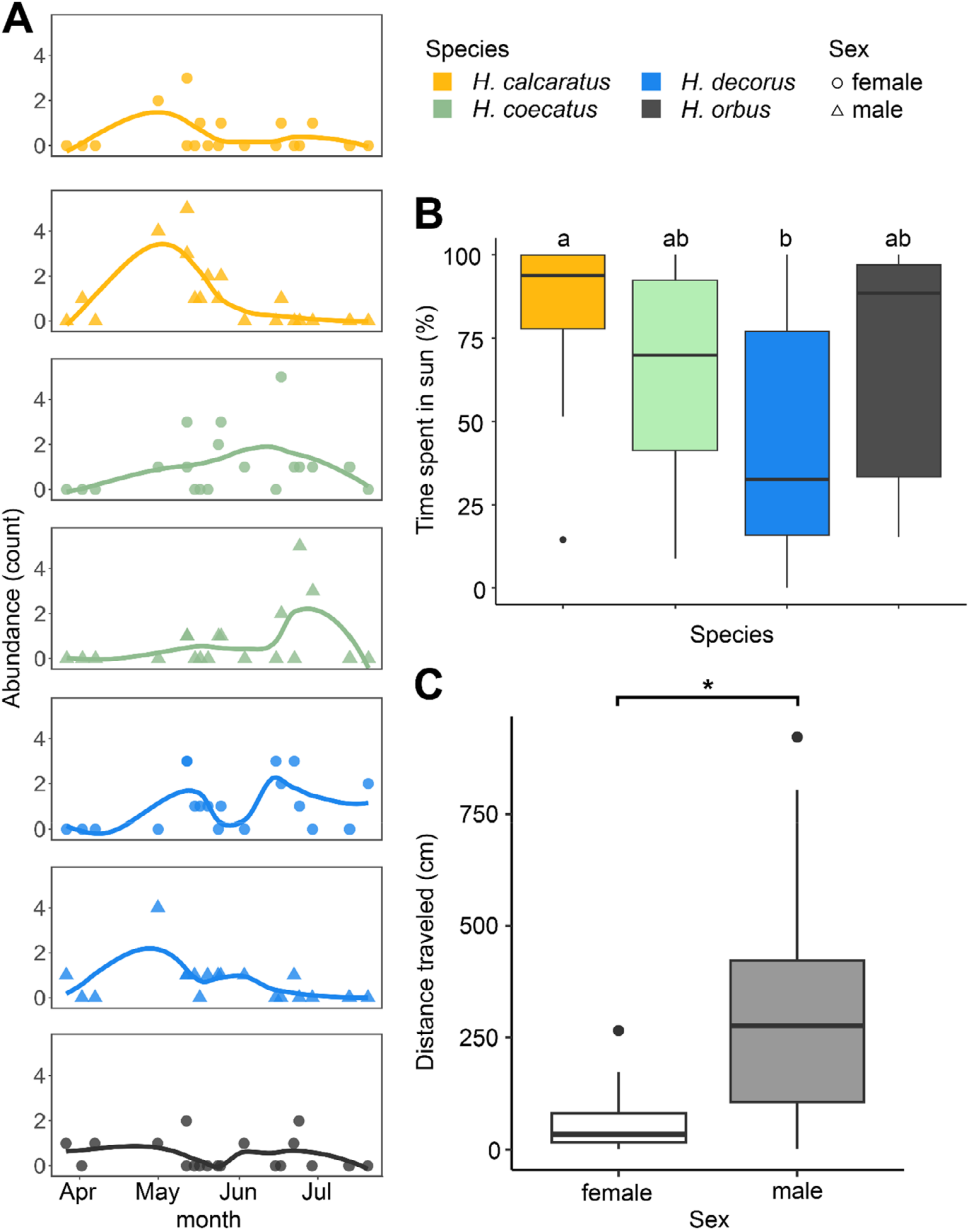
Spider phenology, light environment, and movement activity through the habitat. Abundance of each species and sex during the active season (A). Colors denote species identity, and shapes denote sex (circle = female, triangle = male). Light environment of each species as shown by proportion of time spent in the sun (B). Movement levels of each sex measured as distance traveled during an observation (C).

Our data showed that mature adults and subadults of all species could be found between April and August (Figure 4A). Of these, *H. calcaratus* were found at peak abundance earlier in the season (early May) while *H. coecatus* was found later in the season (late June). Neither species exhibited a sex difference in seasonal phenology. Females of *H. orbus* and *H. decorus* were found at equal abundance throughout the field season, but *H. decorus* males showed a slight trend to be active earlier in the season (May). In terms of diel activities, all species were found to be active between 930 and 1730 h with no obvious trends in species or sex effects (Figure S3).

### 3.3 Light Environment

Some species differed in their light habitat usage (Figure 4B; Schierer‐Ray Hare test: *H* = 18.22, df = 3, *p* < 0.001; Kruskal–Wallis test: *X* ^2^ = 19.66, df = 3, *p* < 0.001). Specifically, *H. calcaratus* was found significantly more often in the sun than *H. decorus* ( *H. calcaratus* median = 93.8% ± 4.1%, *H. decorus* median = 32.6% ± 6.1%; *Z* = 3.06, *p* = 0.01; Table S1). However, neither sex (*H* = 1.46, df = 1, *p* = 0.23) nor sex by species interaction (*H* = 0.86, df = 2, *p* = 0.65) were found to be significant predictors.

### 3.4 Movement Level

Across all spiders, sex was a significant predictor of movement level (Figure 4C; *H* = 22.4, df = 1, *p* < 0.001), with males traveling significantly greater distances on average than females (male median = 276.3 cm ± 42.6 cm, female median = 34 cm ± 7.0 cm). However, species (*H* = 4.66, df = 3, *p* = 0.20) and the sex by species interaction (*H* = 0.71, df = 2, *p* = 0.70) were not significant predictors.

### 3.5 Behavioral Interactions

In total, 33 pairs of spiders were observed to interact (this represents 54% of all spiders observed near a congener at about 0.5 m or less). Of these interactions, 13 were looking interactions (39%), 14 were courtship interactions (42%), 4 involved aggression (12%), and 2 were other types of behavior (6%). All courtship interactions were solicited by displaying males. In most cases, females would watch from a hiding spot (e.g., under a pebble or stick) or actively evade the male's courtship efforts. No successful mating occurrences were observed. Aggression occurred between a mature male *H. decorus* toward a conspecific male, a mature female *H. coecatus* toward another female *H. coecatus* , and there were two separate occurrences of two mature female *H. coecatus* attacking conspecific males during male courtship displays. The observed aggression between the two *H. decorus* males could represent an instance of competition or territoriality, which is interesting since male *Habronattus* spiders are rarely observed to exhibit intrasexual interactions. For the “other types” of behavior, in the first instance, a *H. coecatus* subadult female attempted to capture a cricket nearby a mature *H. coecatus* female, and both spiders jumped away from each other. In the second case, a mature *H. calcaratus* male pursued a *H. coecatus* subadult male for a short duration.

**FIGURE 5:**
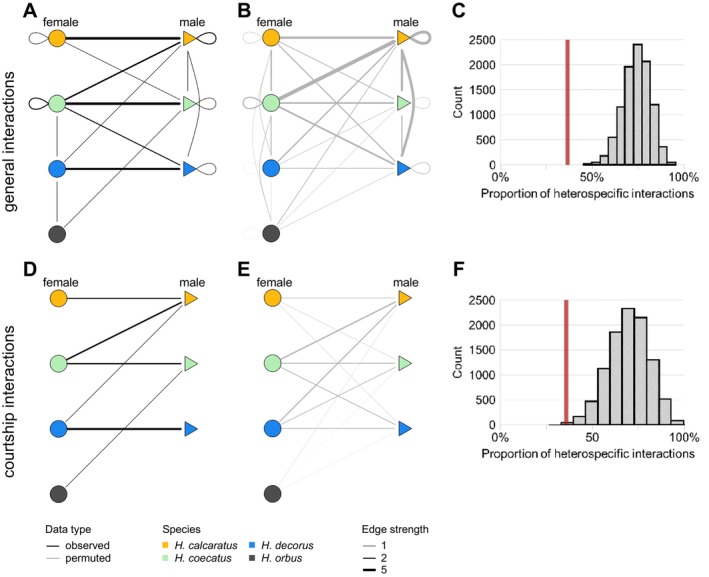
Network plots showing all observed behavioral interactions (A) compared with the average outcome of permuted interactions (B). Distribution of the proportion of heterospecific interactions in the permutation conducted for all interactions (C). The vertical red line denotes the observed percentage of heterospecific interactions (36%). Network plots of observed courtship interactions (D) compared with the average outcome of permuted courtship interactions (E). Distribution of the proportion of heterospecific interactions in the permutation conducted for courtship interactions only (F). The observed heterospecific courtship interaction percentage (36%) is shown by the vertical red line.

In evaluating the intra‐ and interspecific behavioral interactions of the 33 pairs of interacting *Habronattus* spiders, we found that all species and sexes interacted with both conspecifics and heterospecifics (Figure 5A). Heterospecific interactions composed 11% of the total interactions for *H. calcaratus* females; 30% for *H. calcaratus* ; 17% for *H. coecatus* females; 9% for *H. coecatus* males; 50% for both male and female *H. decorus* ; and 100% for *H. orbus* females, the latter due to the lack of conspecific males observed in the area. On the species level, *H. calcaratus* interacted with heterospecifics 30% of the time, 36% for *H. coecatus* , and 38% for *H. decorus* . Compared to our permutation network of general behavioral interactions (Figure 5B), our observed data showed less global, network‐wide heterospecific interactions (36%) than expected by chance at this mixed‐species assemblage ($\bar{x}$ = 74%, *P* < 0.01; Figure 5C). In our observed data, 11 of the 33 spider pairs were the same sex (33%). For the 28 pairs of spiders that were observed near another congener but did not interact, 16 were conspecific pairs (57%) and 12 were heterospecific pairs (43%). 17 of the 28 pairs were the same sex (61%).

When focusing on courtship interactions only, we found that most species and sexes were involved in heterospecific courtships (Figure 5D). *H. calcaratus* males and *H. coecatus* males engaged in courtship with both conspecific and heterospecific females (of which 67% and 25% were heterospecific courtship interactions, respectively), while *H. decorus* only courted conspecific females. For female spiders, *H. coecatus* and *H. decorus* females were courted by both conspecific and heterospecific males (50% and 20% heterospecific courtship interactions, respectively), while *H. calcaratus* females were only courted by conspecific males. The single *H. orbus* female observed to engage in courtship interacted with a heterospecific male (given that conspecific males were not present). On a species level, 50% of interactions were heterospecific for *H. calcaratus* , 40% for *H. coecatus* , and 11% for *H. decorus* . Compared with our permutation results (Figure 5E), this mixed‐species assemblage showed less global heterospecific interactions (36%) as compared to random chance ($\bar{x}$ = 72%, *p* < 0.01; Figure 5F).

## 4 Discussion

We assessed the fine‐scale environmental and behavioral overlaps between four sympatric species of *Habronattus* jumping spiders to explore potential interspecific competition and reproductive interference at this particular mixed‐species assemblage. We observed broad spatial and temporal overlap between all four species at this site (Figures 3 and 4A), suggesting that all species interact with similar parts of the habitat during overlapping seasons. Though we did not estimate population sizes statistically, our observational approach still revealed a slight but notable temporal shift between two species: The peak abundance of *H. calcaratus* was observed earlier in the season around May, whereas the peak abundance of *H. coecatus* was observed later in June (Figure 4A). While *Habronattus* species can be found across diversity environments and habitat types, the spatial and temporal overlaps observed in this study may be attributed to this habitat meeting shared, basic ecological requirements for the species in this assemblage (Bougie, Brelsford, and Hedin 2024; Brandt et al. 2020; Hedin, Foldi, and Rajah‐Boyer 2020; Hedin and Lowder 2009; Hill 2012; Ralston et al. 2017; Taylor, Powell, and McGraw 2017). The spatial and temporal species overlaps may also suggest direct resource competition and reproductive interference between the study species. However, the amount of phenological separation found between *H. calcaratus* and *H. coecatus* in our study has previously been observed to lead to reproductive isolation and sympatric species in closely related species facing direct competition (Bush 1969; Turner and Polis 1979; Uetz 1977). This phenological shift could have arisen through drift, given there is some phylogenetic distance between the two species (Leduc‐Robert and Maddison 2018; Maddison and Hedin 2003), or dietary partitioning, where two species diverge in their preferred prey items that themselves show a difference in seasonal abundance (e.g., Reid et al. 2013). However, the latter is unlikely since *Habronattus* spiders often feed on diverse prey types (personal observations, JYS; Taylor, Clark, and McGraw 2011). Alternatively, the temporal shift may reduce interspecific encounters and behavioral interactions, which has been observed between related to even cause allochronic isolation (e.g., in insects, spiders, birds, fish, and corals; Rohde et al. 2017; Sirkiä et al. 2018; Taylor and Friesen 2017; Uetz 1977).

This pattern may potentially be most applicable to *H. orbus* and *H. calcaratus* , the two closest related species in this assemblage. If *H. orbus* and *H. calcaratus* males equally pursue conspecific and heterospecific males, then the complete absence of *H. orbus* males from the study site could suggest an adaptation to reduce reproductive interference between the two species or *H. orbus* males being outcompeted. However, follow‐up studies should first address whether the absence of male *H. orbus* was due to undersampling. Males may occupy substrate types such as leaf litters that were rare or absent from the study site (Wayne P. Maddison, personal communications). Future studies can also sample multiple sites to examine whether *H. calcaratus* consistently outcompetes *H. orbus* and which factors may affect the outcome of competition (e.g., Lackey et al. 2024; Vági and Hettyey 2016). Additionally, behavioral experiments may reveal the potential role of sexual selection for certain male courtship display elements and/or behavior in driving spatiotemporal divergence (e.g., Elias, Hebets, and Hoy 2006; Hebets and Maddison 2005; Masta and Maddison 2002). The absence of *H. orbus* males also highlights intersexual differences in the abundance and/or habitat usage between males and females, which could be driven by intersexual competition and/or reflect intersexual differences in reproductive strategies (e.g., Breed et al. 2006; Hochkirch, Gröning, and Krause 2007).

Next, we examined fine‐scale microhabitat overlaps between species at this assemblage by comparing their light environment. We found gross overlaps in the light environment occupied by each species, with the exception that *H. decorus* spent significantly less time in the sun than *H. calcaratus* (Figure 4B). Additionally, while there was broad spatial overlap between *H. decorus* and the other three species, *H. decorus* was often found at the perimeter of its spatial distribution, perhaps indicating a preference for forest edges (Figure 2). Together, these suggest that *H. decorus* may occupy a microhabitat unique from the other species (i.e., low light‐level environment shaded by grass and foliage). Notably, unlike males of the other species at the assemblage, *H. decorus* males have abdominal structural coloration that is prominently displayed in a raised position during courtship (Blackwall 1846; Elias et al. 2012; Peckham and Peckham 1883; personal observations, JYS). Being the most phylogenetically distant species within this assemblage, these difference in microhabitat, morphology, and behavioral may have evolved in *H. decorus* through neutral evolutionary processes (Vellend 2010). Alternatively, this could be an adaptation to enhance signaling efficiency in its light‐limited environment by increasing reflectance and/or providing polarization cues. The use of structural coloration and polarized light in signaling has evolved under various contexts across different animal lineages, including butterflies, cuttlefish, mantis shrimp, fish, birds, crabs, flies, and spiders (Cronin et al. 2003; Foelix, Erb, and Hill 2013; Gagnon et al. 2015; Marshall et al. 2019; Shashar, Rutledge, and Cronin 1996; Stuart‐Fox et al. 2021) and may be favored in low light habitats (Douglas et al. 2007). However, the low light environments examined in this study was defined by shadows of local flora, and future studies may rather examine effects of sunny vs. overcast conditions, which can more drastically affect visual signal transmission and perception (Cronin et al. 2014). Other types of microhabitats that were not quantified in this study may also be important to consider. For example, substrate type has been shown in different spider lineages to affect vibratory signals and may lead to behavioral isolation between species (Choi, Miller, and Hebets 2024; Rosenthal et al. 2019; Stratton and Uetz 1983; Sun et al. 2021).

We also examined how species may engage with the environment differently by quantifying their movement activity through the habitat. While there were no species differences, we found that males moved through the environment significantly more than females. This may reflect a male “scramble competition” strategy that is common to all species in this assemblage, where males are under selection to move quickly through the environment to increase their encounter rate with prospective female mates (see Herberstein, Painting, and Holwell 2017). This is consistent with previous characterizations of *Habronattus* mating systems, in which males actively seek potential mates and perform elaborate and species‐specific courtship displays with multimodal components that can serve as the basis for female mate choice (Blackburn and Maddison 2015; Brandt et al. 2020; Elias et al. 2012; Scheidemantel 1996; Taylor, Cook, and McGraw 2019; Taylor and McGraw 2013).

Lastly, we examined direct evidence of interspecific interactions amongst the species at this assemblage. We found that species in this assemblage readily engaged in heterospecific interactions (Figure 5A), including heterospecific courtships (Figure 5D). *H. calcaratus* and *H. coecatus* males engaged heterospecific females in courtship, while *H. decorus* males only courted conspecific females despite encounter a low level of heterospecific females. This could again be a phylogenetic signal and/or the product of species differences in light environment microhabitats, since *H. decorus* was found more often in shaded environments, or there may be some level of reinforcement selection for increased mating success between conspecific *H. decorus* , perhaps related to the usual courtship display and behaviors of *H. decorus* male. Behavioral experiments manipulating different male courtship behaviors and traits may help disentangle the drivers of reproductive isolation in these species. During our observation, not all spiders interacted with congeners that were in close proximity. Interestingly, when we compared spider pairs that interacted vs. pairs that did not interact, we saw that 67% of spider pairs that interacted were different‐sex pairs, while 39% of spiders pairs that did not interact were different‐sex pairs. Future studies may examine whether this pattern can be observed on the populational level, whether it is driven by male behavior, and what mechanisms may be selecting for increased interactions between sexes.

In our observations, no successful copulation was observed for any courtship interaction, regardless of whether courtship occurred between conspecific or heterospecific pairs. This observation is consistent with previous field observations of low mate acceptance rates by *Habronattus* females, even in conspecific pairings and denser populations (0%–7%; Blackburn and Maddison 2015; Scheidemantel 1996; Taylor, Powell, and McGraw 2017). The rarity of observable successful mating is likely due to female *Habronattus* spiders commonly mating only once, compounded with the numerous opportunities for females to encounter males (Blackburn and Maddison 2015; Miller 1971; Scheidemantel 1996). Additionally, laboratory experiments using *H. coecatus* from the Edge of Appalachia showed less than 50% copulation rate even in naïve, unmated females (Sung 2024). These experiments were conducted within a finite space, where females could not fully remove herself from courting males. Comparatively, females in the field can easily escape from unwanted mating attempts, consistent with the low copulation rate observed in this study. Generally, the “choosiness” in *Habronattus* females may be a selected trait to reduce potential energetic costs in engaging with males, including both precopulatory and postcopulatory reproductive investments (Blackburn and Maddison 2015; Herberstein, Painting, and Holwell 2017).

Overall, our observations of heterospecific courtship interactions suggest that species in this assemblage may experience some level of reproductive interference, similar to the findings for another mixed‐species *Habronattus* assemblage (Taylor, Powell, and McGraw 2017). However, like Taylor, Powell, and McGraw (2017), we found that behavioral interactions in our assemblage were biased significantly toward conspecifics (Figure 5C,F). One potential explanation may be that there is a high cost for heterospecific courtship and interaction (e.g., predation of males by females) that selects against interspecific interactions. Conversely, there may be strong selection for females but not males to mate with conspecific individuals, since many males in our study appeared willing to initiate courtship with heterospecific females. No hybrid individuals were observed at this site, though phylogenetic, behavioral, and morphological evidence would suggest that hybridization is possible, given that introgression has been found at substantial levels within the genus (Bougie, Brelsford, and Hedin 2024; Hedin and Lowder 2009; Leduc‐Robert and Maddison 2018; Maddison and McMahon 2000). Whether misdirected courtship and potential reproductive interference results in fitness costs to species in this assemblage remains to be clarified by additional studies. For example, it is possible that the energetic expenditure of unsuccessful courtship could be a negligible cost to the fitness of the male (Barske et al. 2014). Additionally, calculating populational estimations for our observations may further clarify whether the observed patterns and rates of behavioral interactions reflect low populational density of spiders at the site or an underestimation of the population.

Altogether, our findings of the overall overlaps between species in their spatial, temporal, light environment, movement activity, and behavioral interactions suggest that individuals within this mixed‐species assemblage likely face a mix of both interspecific competition and reproductive interference. However, there is also evidence for evolutionary adaptations to alleviate these pressures (e.g., temporal separation between *H. coecatus* and *H. calcaratus* , the spatial and light environment differences for *H. decorus* ). This assemblage may therefore be in the process of evolving under reinforcement selection between syntopic species to reduce competition and reproductive interference. While the specific relationships within this assemblage observed in this study are not directly translatable to other populations and assemblages, our study provides important inferences on the potential conflicts and interference that may arise during direct and indirect interspecific interactions. The extensive geographical distribution of *Habronattus* species across the Americas (Griswold 1987; Richman, Cutler, and Hill 2012) offers exciting opportunities to examine these patterns in other mixed‐species assemblages of *Habronattus* spiders, with different or similar species composition and environmental factors. Such comparative studies would contribute to ongoing efforts across broad taxonomical lineages toward understanding the phylogenetic and environmental drivers of species competition, reproductive interference, and coexistence (see Weber and Strauss 2016).

Furthermore, our study establishes the ecological foundation to examine signal evolution in *Habronattus* jumping spiders. For example, how do signals evolve based on the environment (i.e., under sensory drive hypothesis; Endler 1992)? Factors such as substrate type and thermal microclimates have been found to affect signal transmission and mating success in jumping spiders and wolf spiders (Brandt, Kelley, and Elias 2018; Brandt, Rosenthal, and Elias 2020; Hebets et al. 2008; Stratton and Uetz 1983; Sun et al. 2021). Sexual selection may also affect signal evolution, for example through reproductive character displacement, which can occur in different sensory modalities, such as visual and acoustic (Allen, Stevens, and Higham 2014; Höbel and Gerhardt 2003; Kirschel, Blumstein, and Smith 2009; Marshall and Cooley 2000; Tynkkynen, Rantala, and Suhonen 2004). An interesting visual center to examine reproductive character displacement is the face, which has evolved to facilitate social interaction across diverse lineages (e.g., mammals, fish, bird, spiders, wasps; Harland and Jackson 2000; Leopold and Rhodes 2010; Parr et al. 2000; Sheehan and Tibbetts 2011; Siebeck et al. 2010; Winters, Allen, and Higham 2020). In *Habronattus* jumping spiders, males have species‐specific patterns on its clypeus, or “face” (Figure 1; Elias et al. 2012; Griswold 1987), yet we are only beginning to understand the role that faces may play in intra‐ and interspecific communication in *Habronattus* spiders and how they may have diversified (Sung 2024; Taylor and McGraw 2013).

Ultimately, our field‐based approach revealed interspecific dynamics within a mixed‐species assemblage of diverse jumping spiders on a behaviorally relevant scale, offering insights into potential reproductive interference and critical natural context to inform subsequent laboratory‐ and field‐based studies (see Gröning and Hochkirch 2008). As outlined by Münkemüller et al. (2020), detailed observational studies remain an integral part in formulating and refining both experimental and modeling studies, and it is the combination of all three methodologies that most effectively deepen our understanding of the ecological mechanisms that shape the interactions between communities, species, and their environments.

## Author Contributions

**Jenny Y. Sung:** conceptualization (equal), data curation (lead), formal analysis (lead), funding acquisition (supporting), investigation (lead), methodology (lead), project administration (lead), supervision (lead), visualization (lead), writing – original draft (lead), writing – review and editing (lead). **Jack A. Fogle:** data curation (supporting), formal analysis (supporting), funding acquisition (supporting), investigation (supporting), methodology (supporting), writing – review and editing (supporting). **Nathan I. Morehouse:** conceptualization (equal), funding acquisition (lead), project administration (supporting), resources (lead), supervision (supporting), writing – review and editing (supporting).

## Conflicts of Interest

The authors declare no conflicts of interest.

## Supporting information


Data S1.

## Data Availability

The data used for this study are available at https://datadryad.org/stash/share/Dr2p_b1agB9FF7rwIyV_hZaO9Mm2giwF‐oufuu6I5hM.
