# Supplementary material for: Spatial Overlap and Behavioral Interactions Among Four Habronattus Jumping Spider Species in a Mixed‐Species Assemblage
Source: Ecol Evol. 2025 Mar 30;15(4):e70871. doi: 10.1002/ece3.70871 (PMC11955511; doi:10.1002/ece3.70871)
Supplement: Supplementary file 1 — Data S1. [file ECE3-15-e70871-s001.docx]

# Supporting information


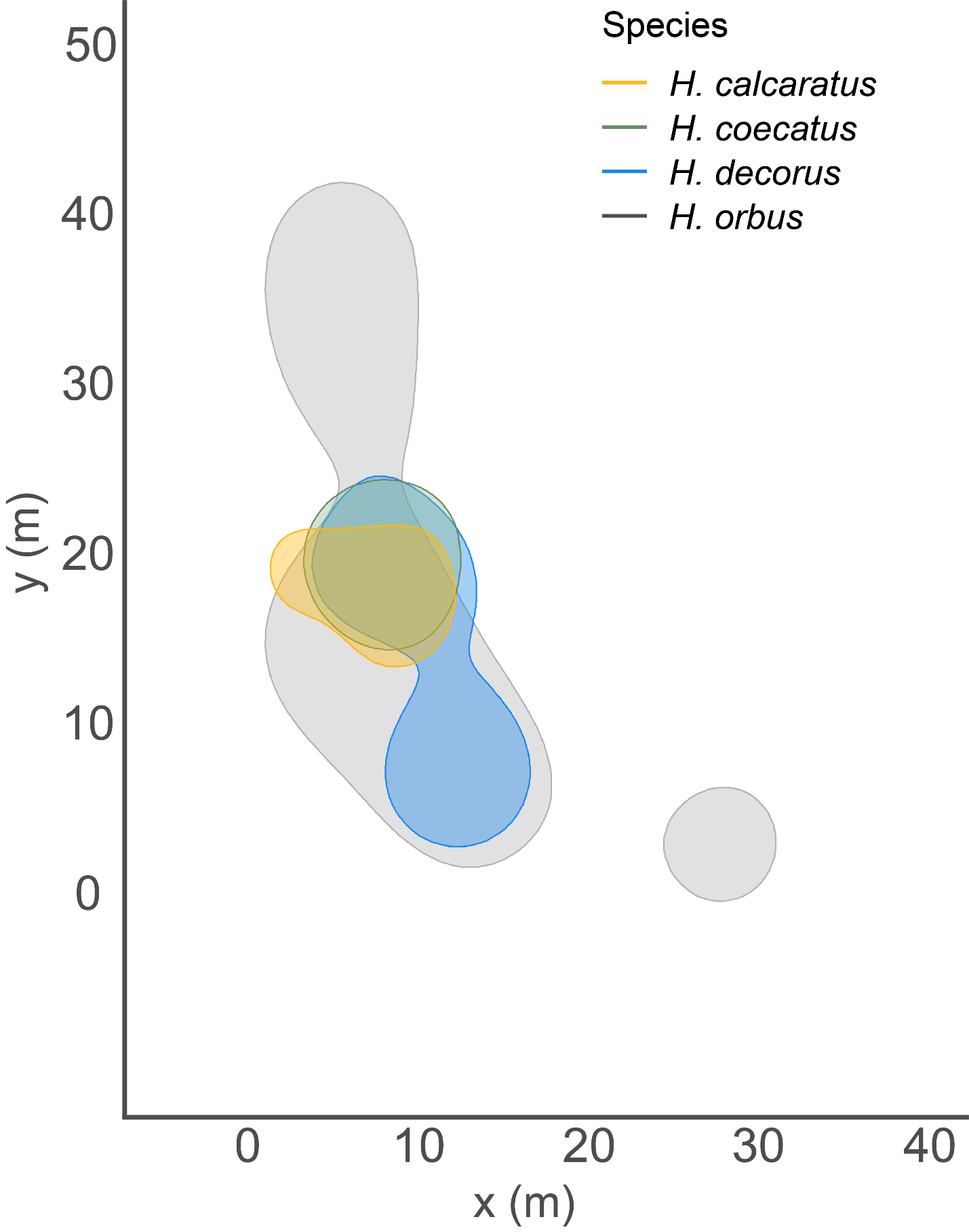


**Figure S1.** Core home range (estimated 50% space usage) of all four species. Color represents species.

**
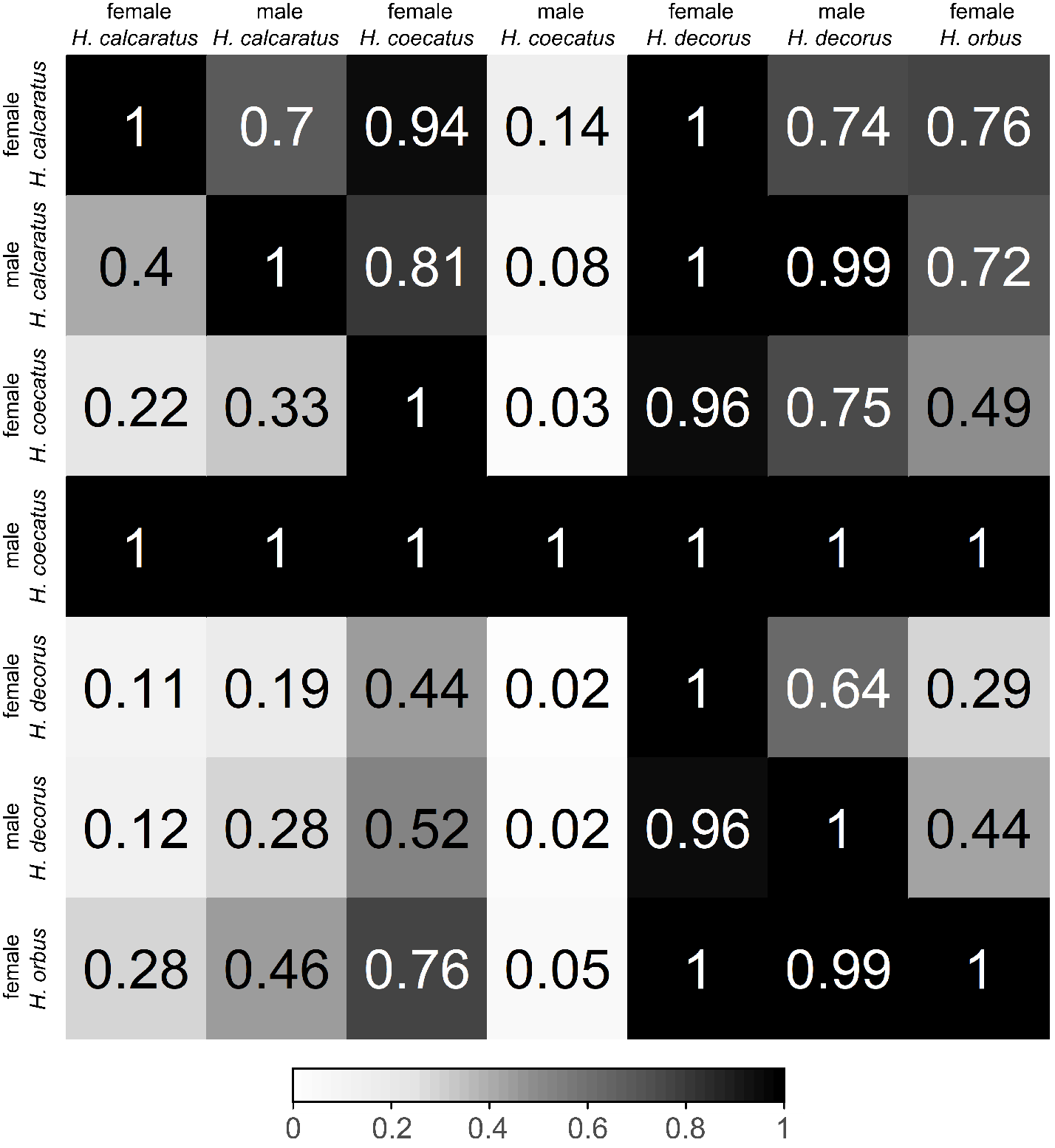
**

**Figure S2.** Overlaps calculated for the extended home range (95% KUD estimation) between each pair of species/sex. Overlap values were calculated by the amount overlap between the species-sex pair divided by the total home range of the species represented in the row (overlap*_i_*_,_*_j_* / HR*_i_*). A value of 1 denotes complete overlap, and 0 denotes no overlap.


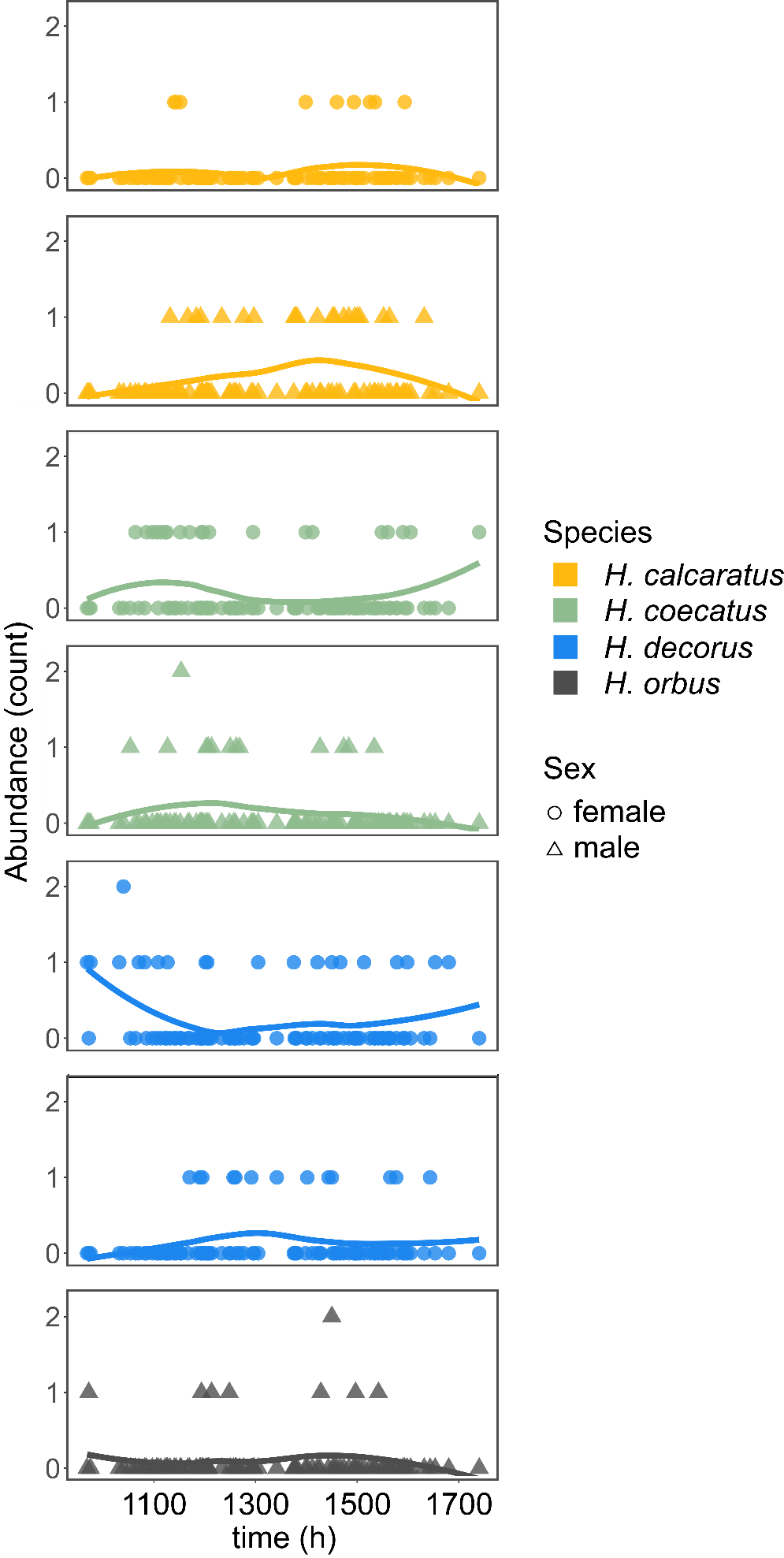
**Figure S3.** The time of day when spiders were found. Color represents species, and shapes represent sex (circle = male, triangle = female).

**Table S1.** Light habitat preference comparison using the Dunn’s Kruskal-Wallis Multiple Comparisons test with adjusted p-values**.**

| Species pair comparison | Z | *p* |
| --- | --- | --- |
| *H. calcaratus - H. coecatus* | 2.37 | 0.11 |
| *H. calcaratus - H. decorus* | 4.41 | **>0.01** |
| *H. calcaratus - H. orbus* | 1.03 | 1.00 |
| *H. coecatus - H. decorus* | 1.96 | 0.30 |
| *H. coecatus - H. orbus* | -0.44 | 1.00 |
| *H. orbus - H. decorus* | 1.61 | 0.64 |
